# The effectiveness and safety of treatments used for acute diarrhea and acute gastroenteritis in children: protocol for a systematic review and network meta-analysis

**DOI:** 10.1186/s13643-016-0186-8

**Published:** 2016-01-20

**Authors:** Ivan D. Florez, Reem Al-Khalifah, Javier M. Sierra, Claudia M. Granados, Juan J. Yepes-Nuñez, Carlos Cuello-Garcia, Giordano Perez-Gaxiola, Adriana M. Zea, Gilma N. Hernandez, Areti-Angeliki Veroniki, Gordon H. Guyatt, Lehana Thabane

**Affiliations:** Department of Clinical Epidemiology & Biostatistics, McMaster University, Juravinski Site. G Wing, 2nd Floor; 711 Concession Street, Hamilton, ON L8V 1 C3 Canada; Department of Pediatrics, Universidad de Antioquia, Medellín, Colombia; Department of Pediatrics, King Saud University, Riyadh, Saudi Arabia; Department of Pediatrics, Division of Endocrinology and Metabolism, McMaster University, Hamilton, Canada; Department of Clinical Epidemiology & Biostatistics, Pontificia Universidad Javeriana, Bogotá, Colombia; Hospital Pediatrico de Sinaloa, Culiacan, Mexico; School of Nutrition and Dietetics, Universidad de Antioquia, Medellín, Colombia; Knowledge Translation Program, Li Ka Shing Knowledge Institute, St. Michaels Hospital, Toronto, Canada; Department of Medicine, McMaster University, Hamilton, Canada; Department of Pediatrics and Anesthesia, McMaster University, Hamilton, ON Canada

**Keywords:** Diarrhea, Gastroenteritis, Children, Zinc, Probiotics, Smectite, Racecadotril, Yogurt, Lactose intolerance, Network meta-analysis, Systematic review

## Abstract

**Background:**

Acute diarrhea and acute gastroenteritis (AD/AGE) are common among children in low- and middle-income countries (LMIC) and high-income countries (HIC). Supportive therapy including maintaining feeding, prevention of dehydration, and use of oral rehydration solution (ORS), is the mainstay of treatment in all children. Several additional treatments aiming to reduce the episode duration have been compared to placebo, but the differences in effectiveness among them are unknown.

**Methods and analysis:**

We will conduct a systematic review of all randomized controlled trials evaluating the use of zinc, vitamin A, probiotics, prebiotics, synbiotics, racecadotril, smectite, and fermented and lactose-free milk/formula for AD/AGE treatment in children. The primary outcomes are diarrhea duration and mortality. Secondary outcomes are diarrhea lasting 3 or 7 days, stool frequency, treatment failure, hospitalizations, and adverse events. We will search MEDLINE, Ovid EMBASE, CINAHL, the Cochrane Central Register of Controlled Trials (CENTRAL), and LILACS through Ovid, as well as grey literature resources. Two reviewers will independently screen titles and abstracts, review full texts, extract information, and assess the risk of bias (ROB) and the confidence in the estimate (with the grading of recommendations, assessment, development, and evaluation [GRADE] approach). Results will be summarized narratively and statistically. Subgroup analysis according to HIC vs. LMIC, age, nutrition status, and ROB is planned. We will perform a Bayesian network meta-analysis to combine the pooled direct and indirect treatment effect estimates for each outcome, if adequate data is available.

**Discussion:**

This is the first systematic review and network meta-analysis that aims to determine the relative effectiveness of pharmacological and nutritional treatments for reducing the duration of AD/AGE in children. The results will help to reduce the uncertainty of the effectiveness of the interventions, find knowledge gaps, and/or encourage further research for other therapeutic options.

**Systematic review registration:**

PROSPERO registration number: CRD42015023778.

## Background

Diarrheal diseases remain the third cause of death among children younger than 5 years of age [1, 2]. Almost all of these deaths occur in low- and middle-income countries (LMIC). Although in high-income countries (HIC) the disease is rarely fatal, it is a leading cause of emergency department visits and hospitalizations [3].

Acute diarrhea (AD) is defined by the World Health Organization (WHO) as the passage of three or more loose or liquid stools per day, for three or more days and for less than 14 days [4]. On the other hand, the American Academy of Pediatrics (AAP) defines acute gastroenteritis (AGE) as the diarrheal disease of rapid onset, with or without additional symptoms and signs, such as nausea, vomiting, fever, or abdominal pain [5]. The AAP restricts this definition to children 1 month to 5 years of age who live in developed countries and to episodes of less than 10 days of duration [5]. Although AGE and AD are supported in two different definitions, they are usually related to the same disease, a gastrointestinal infection caused by microorganisms such as rotavirus, *Salmonella*, *E. coli*, and *Campylobacter*, among others [6]. Nevertheless, the pattern of etiology is variable, given that in HIC, the predominant causes are usually viruses, while in LMIC, the bacterial microorganisms play a more important role.

AGE is the preferred term in HIC, where it is considered a self-limited disease with some potential of causing hospitalization for a few days. Meanwhile in LMIC, the most common name used is AD, and it is more frequently associated with a prolonged disease with the potential of causing severe dehydration, malnutrition, and death. These differences explain why there are different scopes in the literature in terms of definitions as well as in terms of treatment recommendations. According to the WHO, the mainstay of AD’s treatment is the prevention of dehydration with adequate liquids and oral rehydration salts (ORS), maintenance of oral feeding, and supplementation of zinc [4]. In HIC, although the ORS and oral feeding maintenance are also the main recommendations, some guidelines have either recommended additional treatments such as probiotics, racecadotril, and smectite and/or have discouraged the use of zinc [7, 8].

Efforts in LMIC have been made to reduce mortality by maintaining the oral feeding and increasing the ORS and zinc use, which could reduce diarrhea and has some nutritional and immunological advantages [9]. Also, supplementation with vitamin A and micronutrient mixtures have been tested for improving different outcomes in AD in the LMIC setting [10]. On the other hand, in HIC, malnutrition and mortality not being real problems, the efforts have focused on reducing the days of diarrhea with the pharmacological interventions. Furthermore, in addition to the micronutrients and pharmacological treatments, other interventions have been used to reduce the duration of the disease and the rates of hospitalizations, such as fermented milks (i.e., yogurt, kefir, or kumis) and the lactose-free formulas [11, 12].

Trials comparing different interventions have been carried out in both settings. In comparison to placebo, most of these interventions have been found effective, an evidence that has been synthesized through some systematic reviews [11, 13–19], while other reviews are still ongoing [20, 21]. However, differences seem to exist in the effectiveness of intervention based on the country setting of the studies. For instance, zinc trials have been mostly conducted in LMIC, where it was shown to be effective against placebo [13], and zinc is recommended by the WHO [4] mostly focused on the LMIC, while it is not routinely recommended in HIC [8]. Meanwhile, the trials using pharmacological treatments against placebo have been conducted in both HIC and LMIC [15].

As a result, to date, although some interventions have been shown to be effective, it is not clear whether the children should be treated equally regardless of the country setting or the nutrition status, and neither is it clear which one of the interventions is better than the others. Only one recent systematic review performed direct and indirect comparisons and found that racecadotril was the best available option for reducing the diarrhea duration [22]. However, this review having included only three interventions and probiotics, provided limited information about search strategies, included and excluded studies, and the risk of bias (ROB) assessment, it did not assess for potential effect modifiers and the overall confidence in the estimate, and it was published in Spanish.

To date, there is no systematic review and network meta-analysis (NMA) that has compared all the available standard treatments for AD/AGE in children. Therefore, the aim of this project is to assess the effectiveness and safety of the different treatments, in addition to the oral rehydration for AD/AGE in children in relation to one another, based on the specific country setting, through direct and indirect comparisons using a Bayesian approach, in order to enhance current treatment of the disease.

## Methods and design

This systematic review and NMA protocol has been registered in the PROSPERO international prospective register of systematic reviews (CRD42015023778), and it was developed following the preferred reporting items for systematic review and meta-analysis protocols (PRISMA-P) guidance [23]. The final report will comply with the recommendations of the PRISMA extension statement for reporting of systematic reviews incorporating network meta-analyses of health care interventions [24].

### Data sources and search strategy

Literature searches will be performed in MEDLINE, EMBASE, Global Health through the Ovid platform, and also in CINAHL, LILACS, and the Cochrane Central Register of Controlled Trials (CENTRAL) using a combination of controlled and free-text terms with various synonyms for the disease and the interventions. The example of the search strategy through Ovid MEDLINE is shown in Appendix A: Table 2. We will use the validated randomized controlled trial (RCT) filters created by the McMaster University Health Information Research Unit for MEDLINE and EMBASE through the Ovid platform. These filters provide a good balance between sensitivity and specificity [25]. In addition, we will use previously validated and used filters for identifying pediatric articles in MEDLINE (translated to the other databases through Ovid), CINAHL, and CENTRAL [26–28].

Search alerts will be set up for monthly notification, and the search will be repeated before the final manuscript submission. Search strategies were developed with liaison with an experienced librarian. No language, publication status, or date limit will be used.

We will carry out a manual hand search of bibliographies of identified RCTs and guidelines. Additionally, we will perform a grey literature search through (1) trial registries (e.g., clinicaltrials.gov and the WHO International Clinical Trials Registry Platform Search Portal), (2) conference proceedings and abstracts, and (3) dissertation databases (ProQuest Dissertations and Theses database). We will contact authors of unpublished work to ensure eligibility.

### Eligibility criteria

The search for studies will be limited to RCTs assessing the efficacy, effectiveness, or safety of different interventions for AD/AGE in children younger than 18 years of age. The diarrhea episode will have to be proven or presumed to be caused by an infectious agent, with a duration of less than 14 days. Diarrhea is usually defined as three or more loose stools in a 24-h period [4, 5].

We will include RCTs and quasi-RCTs that evaluated single and/or combined interventions at any dose and presentation of zinc, vitamin A, micronutrients, smectite, racecadotril, prebiotics, probiotics, synbiotics, fermented milks, and lactose-free milks and formula. Regarding probiotics, we will search for trials using, but not restricted to: *Lactobacillus* or *Bifidobacterium* strains, *Sacharomyce*s *boulardii*, *Bacillus clausii* or *subtilis*, *Streptococcus thermophylus*, and *Enterococcus faecium*, or mixtures of two or more probiotics. Regarding the prebiotics, we will search for trials studying inulin, fructo- and galacto-oligosaccharides. We will consider a synbiotic when a pharmacological preparation contains one or more probiotics and one or more prebiotics.

Regarding fermented milks, we will include studies using preparations based on *S. thermophilus* or *L. delbrueckii* subsp. *bulgaricus* cultures such as kefir, kumis, and yogurt. Fermented milks supplemented with probiotics will be also included. Finally, as to lactose-free milks, we will include studies evaluating as interventions lactose-free, or diluted (by at least 50 %) milks or formula, including soy-based milks.

We will not include studies testing the effectiveness of antimicrobials for bacterial or parasitic infections because these treatments are not empirically recommended and are only prescribed under certain clinical conditions or for a specific microorganisms’ detection. We will not include neither studies testing bismuth nor diphenoxylate since these drugs are not approved for their use in young children. Lastly, we will exclude studies comparing alternative or non-mainstream interventions, given that we are only interested in widely available treatments approved for their use in children (standard treatments), and such treatments are out of the scope of our question.

The studies will have to compare any of these interventions; among them are different doses of the same intervention, or with placebo, or no intervention (conventional treatment with ORS). In order to be included, the study will have to report the effectiveness of one or more of the described interventions with at least one of the outcomes of interest.

Our primary outcomes are mean diarrhea duration (hours/days) and mortality. The secondary outcomes include: diarrhea lasting >3 or 7 days, stool frequency, diarrhea hospitalizations, treatment failure, and adverse events (vomiting, somnolence/lethargy, ileus, constipation). Table 1 describes the definitions of the outcomes. We will exclude studies in patients with cholera, HIV, or with known or thought to have other causes of diarrhea such as antibiotic-associated, persistent or chronic diarrhea. In case the trials have included children with some of the exclusion criteria, and the authors report the results separately, we will use the specific results for our population of interest. Otherwise, we will include the study if we have information about the proportion of children with the exclusion criteria, and this is less than 30 % of the total sample size.

**Table 1:** Outcome measures

Outcomes	Measurement of variables (units)	Statistical estimates and measurement of association
Primary		
Mean diarrhea duration	Duration in hours/days	MD (95%CrI)
Mortality	Number of deaths	OR (95%CrI)
Secondary		
Diarrhea lasting >3 or 7 days	Number of children with diarrhea >3 and 7 days of duration	OR (95%CrI)
Stool frequency at third day	Number of stools/day at third day	MD (95%CrI)
Diarrhea hospitalizations	Number diarrhea-related hospitalizations	OR (95%CrI)
Treatment failure	Number of children with treatment failure	OR (95%CrI)
Adverse events	Side effects:	OR or MD (95%CrI)
	Vomiting	
	Abdominal pain	
	Somnolence/lethargy	
	Ileus	
	Constipation	

*MD* mean difference, *OR* odds ratio, *95%CrI* 95 % credibility interval

### Study selection

Paris of reviewers (IDF, CMG, CCG, JMS, AMZ, GPG, JJY, and RA) will perform, independently and in duplicate, the screening of available titles and abstracts to assess its eligibility. As a second step, the full-text articles of the potentially eligible studies will be screened to assess their eligibility. We will include studies where both reviewers agree about their inclusion. In case of disagreement between the reviewers, a third member (IDF, CMG, or JMS) of the team will resolve it. Records of excluded articles in this stage, along with the reason for ineligibility, will be saved for future reference. Reviewers will refer to the eligibility criteria during the screening process. Eligible article citations will be saved in the Endnote X7 library. We will present the PRISMA flow diagram [29], demonstrating the search and screening process. We will contact authors of primary studies during data extraction to obtain any missing information.

### Data extraction

The study data will be collected based on a pre-specified instruction extraction form in a Microsoft Excel sheet form. We will extract the following information: study characteristics (design, year, duration of follow-up, sample size per arms, setting, and country), patient characteristics (age, inpatient/outpatient, nutrition status, days of disease, etiology, and hydration status), intervention details (doses, administration forms), and outcome results (number of events, mean and standard deviation or standard errors per arm) at the longest duration of follow-up. All reviewers will test the data extraction form before the review. Eight reviewers (IDF, CMG, CCG, JMS, AMZ, GPG, JJY, and RA) will perform data extraction, working in pairs independently and in duplicate. If a consensus cannot be reached, a third designated reviewer will be involved (IDF, CMG, or JMS).

### Assessment of risk of bias in included studies

We will assess all included studies for their ROB. For this purpose, two independent pairs (IDF, CMG, CCG, JMS, AMZ, GPG, JJY, and RA) will use a modified version of the Cochrane ROB tool [30] based on sequence generation, allocation concealment, blinding of participants, personnel and outcome assessors, completeness of follow-up, selective outcome reporting, and other biases. Each criterion will be assigned a score of “definitely low risk”, “probably low risk”, “probably high risk”, or “definitely high risk” [31]. We will try to resolve by consensus the disagreements between two reviewers when assessing the studies. Nevertheless, if a consensus cannot be reached, a third designated reviewer (IDF or GHG) from the team will be involved to resolve it.

### Direct comparisons and assessment of heterogeneity

We will first describe the results narratively and, where possible, the direct evidence will be pooled. Given that we expect clinical and methodological heterogeneity among the studies (see below in the Rating the confidence in estimates of the effect in NMA section), which in turn will create statistical heterogeneity, we will pool direct evidence for each treatment comparison using a random-effects (RE) model. In comparison to a fixed-effect model (FE), the RE model is conservative in that it considers both within- and among-study variability. The RE models assume that the observed treatment effect for a study is a combination of a treatment effect common to all studies plus a component specific to that study alone [32]. We will pool the outcome data using a Bayesian RE model [33]. Effect estimates along with 95 % credible intervals (CrIs) will be estimated using odds ratio (OR) for binary outcomes, and mean difference for continuous outcomes, if they are reported using the same metrics or standardized mean difference (SMD) otherwise. For studies with binary outcomes, we will add 0.5 to each cell if one arm is zero, whereas we will exclude studies from the analyses with zero events in both arms. We will use non-informative priors for all model parameters apart from the heterogeneity variance parameter, for which we will use the informative prior suggested by Turner et al. [34] and Rhodes et al. [35]. All Bayesian analyses will be performed using the Markov Chain Monte Carlo method.

We will assess heterogeneity by estimating the magnitude of the between-study variance using the empirical distribution as estimated by Turner et al. [34] and Rhodes et al. [35], and by using the *I*^2^ statistic to quantify the percentage of variability that is due to true differences between studies rather than sampling error [36, 37]. We will interpret the *I*^2^ using the thresholds set forth by the Cochrane Collaboration [30], and it will be used as a criterion for pooling the results and for performing additional subgroups analysis. In case there is important heterogeneity, we will use meta-regression to explain it if we have enough data to do so. Otherwise, we will perform subgroup analyses.

We are proposing six a priori hypotheses to explain variability between studies, therefore, as possible effect modifiers; studies in children [1] in HIC could show a smaller effect of micronutrients in comparison to LMIC [2]; micronutrient deficiencies or malnutrition could show a smaller effect of micronutrients in comparison to well-nourished children [3]; a rotavirus infection could have larger effects than those that do not specify the etiology [4]; inpatients could show more larger effects that in outpatients [5]; trials with a higher ROB could show larger effects than trials with a lower ROB; and [6] studies with majority of children younger than 6 months or above 5 years of age could show a smaller effect, mostly of micronutrients, zinc, or vitamin A, but also of the rest of the interventions. We will perform meta-regression using these hypotheses as the study level covariates, and we will perform a sensitivity analysis based on the studies with high a ROB.

The differences among the studies related to the country setting and the nutrition status are closely related. The previously described differences in the burden of the disease in children of LMIC in comparison with children of HIC are related to the higher prevalence of malnutrition and micronutrient deficiencies among the former than the latter. Thus, zinc, vitamin A, and other micronutrients, and also essential nutrients are expected to have a higher effect on reducing the diarrhea in malnourished children than in eutrophic children. However, the lack of information regarding the baseline nutritional or micronutrient status of the children in the studies is a potential problem; therefore, the country setting is an approximation to this source of heterogeneity. We will analyse differences based on both nutritional/micronutrient status and country setting if we have enough data to do so. We will interpret the results of the heterogeneity in the context of the grading of recommendations, assessment, development, and evaluation (GRADE) approach [38].

### Assessment of reporting bias

We will construct a funnel plot for each outcome to assess the potential publication bias [39], if we retrieve at least ten studies [30]. Visual inspection to determine the funnel asymmetry will be used for this purpose. We will also perform the Begg’s rank correlation [40] and Egger’s regression tests [41] if a similar number of studies is available.

### The network meta-analysis: the direct and the indirect evidence

We will perform a random-effects NMA, as we expect between-study heterogeneity. Given that many of the treatment combinations available to treat AD/AGE have not been compared in head-to-head studies, we expect that some of the possible comparisons between the interventions will not have direct evidence. In the absence of direct evidence for a given comparison, the indirect comparison will provide the estimate. In the presence of direct evidence, the NMA will provide a combined estimate (i.e., direct and indirect evidence) [42]. We will combine direct and indirect estimates in a NMA for all interventions on the outcomes if the assumptions of between-study homogeneity and transitivity across treatment comparisons are judged to be justifiable. Violation of the transitivity assumption will cause inconsistency between direct and indirect estimates (loop inconsistency) and/or inconsistency between studies that inform the same treatment comparison but include a different number of treatment arms (design inconsistency). We will apply the design-by-treatment interaction model to evaluate both design and loop inconsistency, and if this suggests inconsistency, then we will apply the loop-specific method to assess local inconsistency [43–45]. We will perform a network meta-regression or subgroup analysis using the same potential treatment effect modifiers described in the “Direct comparisons and assessment of heterogeneity” section to explore important heterogeneity and/or inconsistency. We will also perform a sensitivity analysis for different heterogeneity priors to assess the robustness of results [33, 34, 35].

The network geometry and the results in probability statements as well as forest plots will be presented; this will ensure interpretability of the results. Effect estimates will be presented along with their corresponding 95 % CrIs. We will rank the probabilities with its 95 % CrIs as well as the surface under the cumulative ranking curve (SUCRA) values and rankograms [46]. It is expected that the best treatments will have high SUCRA values while the worst will have low values. For each paired comparison, we will present the direct, indirect, or network estimates.

We will fit a Bayesian hierarchical model with non-informative priors adjusting for correlation of multi-arm trials, assuming a common-within network heterogeneity variance. A series of 100,000 burn-in simulations will be used to allow convergence and then a further 20,000 simulations to produce the outputs. We will assess model convergence on the basis of the Gelman and Rubin diagnostic test [47]. The analysis will be performed in OpenBUGs (version 3.2.3) [48].

### Rating the confidence in estimates of the effect in NMA

Reviewers (IDF, CMG, CCG, JMS, AMZ, GPG, JJY, and RA), in pairs, will independently assess the confidence in the estimates (quality of evidence) for each reported outcome according to the GRADE approach [49]. We will rate the confidence based on four levels: high, moderate, low, and very low. For the direct comparisons, we will assess and rate each outcome based on the following categories: ROB [50], imprecision [51], inconsistency (which is determined based on the heterogeneity as described above) [52], and publication bias [53].

For the assessment of confidence in the estimates, we will use a recent recommended approach [54]. We will assess and rate the confidence in all the indirect comparisons—if available—obtained from first-order loops following the GRADE categories used for assessing the direct comparisons in addition to the transitivity assessment. Transitivity, also called similarity [55], is the assumption that an indirect comparison is a valid method to compare two treatments, because the studies are sufficiently similar in important clinical and methodological characteristics, or in other words, that they are similar in their distributions of effect modifiers [56, 57]. Then, we will rate the confidence in each NMA effect estimate using the higher rating when both direct and indirect evidences are present.

We will assess and rate the confidence in estimates of effect from the direct comparisons in our pair-wise meta-analyses described previously. For rating confidence in the indirect comparisons, we will focus our assessments on first-order loops (FOLs), which are loops connected to the interventions of interest through only one other intervention. For example, if there are interventions A, B, and C, and there are direct comparisons, e.g., A vs. B (AB) and B vs. C (BC), we could indirectly estimate the effects of A vs. C (AC). The AC indirect estimation will be a FOL. We will choose the FOL with the lowest variances, and thus contributes the most to the estimates of effect, for rating the confidence.

Within a FOL, the indirect comparison confidence will be the lowest of the confidence ratings we have assigned to the contributing direct comparisons. For example, if we find that AB has moderate confidence and BC has high confidence, we will judge the associated indirect comparison, AC, as moderate confidence. We may rate down confidence in the indirect comparisons further if we have a strong suspicion that the transitivity assumption has been violated.

Our overall judgement of confidence in the NMA estimate for any paired comparison will be the highest of the confidence ratings among the contributing direct and indirect comparisons. However, we may rate down confidence in the network estimate if we find that the direct and indirect estimates have inconsistency. For this purpose, the GRADE approach recommends to assess the incoherence (or inconsistency as described in the “The network meta-analysis: the direct and the indirect evidence” section) criteria, which is defined as the differences between direct and indirect estimates of effect [54].

## Discussion

Diarrheal diseases remain the major cause for morbidity and mortality among children. This systematic review will provide the relative effectiveness of the treatments for AD/AGE. The results will be of interest for a broad audience: pediatricians, family physicians, general practitioners, guideline developers, policy makers, and researchers, in both LMIC and HIC. To the best of our knowledge, our study will be the first NMA in children to investigate the effectiveness and safety of all the available standard treatments.

Our planned methods for this review have many strengths. First, we will implement a wide-search strategy that included published work in six different databases, as well as unpublished work. Second, we aim to report important outcomes in children. Third, our study will take into account the GDP setting and some other sources of heterogeneity. Therefore, we will provide effect estimates that are relevant to most of the clinical settings. On the other hand, there are some challenges for this review. We anticipate some degree of clinical heterogeneity with regard to the possible sources of heterogeneity that we described. If the extent of included studies is small, the ability to explore heterogeneity could be limited. Finally, we have focused only on the analysis of outcomes that are clinically important and can be studied using NMA in order to produce important information for decision making, in a timely and efficient manner.

We hope that this review will provide evidence to reduce the uncertainty about the ranking of the interventions in terms of effectiveness and safety, improve child health care, will find knowledge gaps, and/or will encourage further research for other therapeutic options.

## Glossary of terms

Direct estimate: an estimate provided by a head-to-head comparison; Indirect estimate: an estimate provided by two or more head-to-head comparisons that share a common comparator (e.g., direct comparisons: AB and BC, indirect estimation: AC); NMA (network meta-analysis); combination of direct (when available) and indirect estimates of a comparison; loops: Two or more head-to-head comparisons that contribute to an indirect estimate. First-order loops (FOLs) are those loops that involve only a single additional intervention; heterogeneity: differences in estimates of effect across studies that assessed the same comparison; inconsistency [54]: the GRADE approach criterion for rating the degree of consistency among the results in the meta-analysis (heterogeneity); incoherence [54]: the GRADE approach term used as criterion for rating the inconsistency, specifically in NMA. It refers to the differences between direct and indirect estimates of effect; intransitivity[54]: differences in study characteristics that may modify treatment effect in the direct comparisons, and could bias the indirect estimate.

## Appendix

**Table 2:** Example of the search strategy for MEDLINE through Ovid

Ovid MEDLINE(R) in-process and other non-indexed citations, Ovid MEDLINE(R) daily, and Ovid MEDLINE(R) 1946 to present	
1. exp Diarrhea/	
2. diarrh$.mp.	
3. exp Gastroenteritis/	
4. gastroenteritis.mp.	
5. gastrointestinal infection$.mp.	
6. enteritis.mp.	
7. dysenter$.mp.	
8. or/1-7	
9. pr$biotic$.mp. or exp Probiotics/	
10. exp Lactobacillus/	
11. lactobacteri$.mp.	
12. lactobacill$.mp.	
13. reuteri.mp.	
14. exp Saccharomyces/	
15. exp Bifidobacterium/	
16. saccharomyc$.mp.	
17. boulardii.mp.	
18. exp Bacillus/ or Bacillus.mp.	
19. Subtilis.mp.	
20. clausii.mp.	
21. exp Enterococcus faecium/	
22. enterococcus faecium.mp.	
23. bifidobact$.mp.	
24. rhamnosus.mp.	
25. casei.mp.	
26. thermophilus.mp.	
27. acidophilus.mp.	
28. plantarum.mp.	
29. bulgaricus.mp.	
30. lgg.mp.	
31. bifidum.mp.	
32. sy?biotic$.mp.	
33. or/9-32	
34. exp Silicates/	
35. silicate$.mp.	
36. diosmectite.mp.	
37. smectite.mp.	
38. smecta.mp.	
39. or/34-38	
40. racecadotril.mp.	
41. acetorphan.mp.	
42. enkephalinase inhibitor$.mp.	
43. exp Thiorphan/	
44. thiorphan.mp.	
45. tiorfan.mp.	
46. tiorfix.mp.	
47. hidrasec.mp.	
48. or/40-47	
49. exp Loperamide/	
50. loperamide.mp.	
51. imodium.mp.	
52. or/49-51	
53. exp Antidiarrheals/	
54. antidiarrh$.tw.	
55. or/53-54	
56. exp Zinc/	
57. exp Zinc Compounds/	
58. exp Zinc Acetate/	
59. zinc.mp.	
60. gluconate.mp.	
61. cinc.mp.	
62. Trace Elements/	
63. or/56-62	
64. exp Vitamin A/	
65. retinol.mp.	
66. vitamin A.mp.	
67. or/64-66	
68. exp Yogurt/	
69. exp Cultured Milk Products/	
70. yogurt$.tw.	
71. yogh?urt$.tw.	
72. k?umis$.tw.	
73. Kumys$.mp.	
74. k?efir$.tw.	
75. kephir.tw.	
76. bulgaricus.tw.	
77. doog.tw.	
78. lassi.tw.	
79. Matso?n$.tw.	
80. Da?hi.tw.	
81. viili.tw.	
82. (Fermented adj2 milk).tw.	
83. delbruecki$.tw.	
84. (sour adj1 milk).tw.	
85. or/68-84	
86. exp Milk Substitutes/	
87. exp Lactose Intolerance/	
88. (Soy adj3 milk).tw.	
89. (Soy adj3 formula).tw.	
90. (lactose adj1 intolerance).mp.	
91. (lactose$ adj2 formula).tw.	
92. or/86-91	
93. 33 or 39 or 48 or 52 or 55 or 63 or 67 or 85 or 92	
94. 8 and 93	
95. randomized controlled trial.pt.	
96. randomized.mp.	
97. blind$.mp.	
98. placebo.mp.	
99. or/95-98	
100. 94 and 99	
101. (Infan$ or newborn$ or new-born$ or perinat$ or neonat$ or baby or baby$ or babies or toddler$ or minors or minors$ or boy or boys or boyfriend or boyhood or girl$ or kid or kids or child or child$ or children$ or schoolchild$ or schoolchild).mp. or schoolchild.tw. or schoolchild$.tw. or adolescen$.mp. or juvenil$.mp. or youth$.mp. or teen$.mp. or under$age$.mp. or pubescen$.mp. or exp Pediatrics/ or pediatric$.mp. or paediatric$.mp. or peadiatric$.mp. or school.tw. or school$.tw. or prematur$.mp. or preterm$.mp.	
102. 100 and 101	
103. limit 102 to human	

## Abbreviations

AD: acute diarrhea
AGE: acute gastroenteritis
DIC: deviance information criterion
GRADE: grading of recommendations, assessment, development, and evaluation
HIC: high-income countries
LMIC: low- and middle-income countries
NMA: network meta-analysis
RCTs: randomized controlled trials
